# Integrating evidence-based health approaches in U.S. healthcare settings: addressing the syndemics of poverty, health, and violence

**DOI:** 10.3389/fpsyt.2026.1788042

**Published:** 2026-04-16

**Authors:** Srishti Meera Sardana, Sydney Timmer-Murillo, Christine L. Larson, Terri A. deRoon-Cassini

**Affiliations:** 1 Department of Psychological & Brain Sciences, University of Wisconsin – Milwaukee, Milwaukee, WI, United States; 2 Division of Trauma & Acute Care Surgery, Principle Investigator, Milwaukee Trauma Outcomes Project, Medical College of Wisconsin, Milwaukee, WI, United States; 3 Department of Psychological & Brain Sciences, University of Wisconsin - Milwaukee, Milwaukee, WI, United States; 4 Division of Trauma & Acute Care Surgery, Medical College of Wisconsin, Milwaukee, WI, United States

**Keywords:** health disparities, integrated behavioral health, social determinants of health, structural inequality, syndemic framework, trauma-informed healthcare, violence and health

## Abstract

Health disparities in the United States are not produced by single risk factors but by interacting social and biological conditions that cluster within structurally marginalized communities. Poverty, violence, and poor physical and mental health form a reinforcing system of disadvantage that traditional healthcare models—organized around isolated diseases—are poorly equipped to address. This perspective examines these dynamics through a syndemic framework, which conceptualizes co-occurring conditions as mutually interacting epidemics intensified by social inequality. Drawing on interdisciplinary evidence from public health, medicine, and social science, we describe how poverty-related stressors such as housing instability, food insecurity, and barriers to healthcare intersect with exposure to interpersonal and structural violence to amplify risks for depression, posttraumatic stress disorder, chronic disease, and premature mortality. These interactions produce compounded health burdens that are disproportionately experienced by marginalized populations. Despite increasing attention to social determinants of health, current healthcare responses remain fragmented. Health systems frequently identify risks through screening for social needs or trauma exposure but lack the institutional infrastructure, reimbursement mechanisms, and cross-sector partnerships required to address them effectively. We argue that advancing health equity requires moving beyond additive models of care coordination toward syndemic-informed healthcare systems that intervene simultaneously on clustered conditions and their shared upstream drivers. We outline key priorities for practice, policy, and research, including linking screening to actionable care pathways, strengthening partnerships between healthcare and social service systems, and expanding workforce training to include structural and syndemic competency.

## Introduction

Poverty and violence are well-known drivers of poor health, yet healthcare systems do not often adapt to meet the needs of those most affected. In the United States (U.S.), more than 37 million individuals live below the federal poverty line, with disproportionately higher rates among Black and Hispanic households, and firearm violence remains a leading cause of death among young people, particularly in urban communities (1, 2). This review examines the intersection of poverty, violence, and physical and mental health in healthcare settings, through a syndemic framework. We focus primarily on healthcare systems in the U.S., particularly urban and structurally marginalized communities where concentrated poverty, exposure to violence, and fragmented health systems intersect. In brief, the syndemic framework is an intentional shift from siloed biomedical models toward a more holistic, contextually grounded understanding of health that accounts for the socio-political determinants underpinning disease clustering, such as the effect of violence and poverty on health outcomes (3). It allows for exploration of evidence-based strategies for integrating health care and outlines an intersectoral team approach to addressing social determinants of health (SDOH) that may amplify health disparities. The viewpoint seeks to highlight the gap in current SDOH screening and discusses systemic barriers, practice guidelines, and policy implications to enhance health systems-based mental health interventions for vulnerable populations.

The interwoven crises of poverty, violence, and poor health—both physical and mental—form a syndemic that disproportionately affects marginalized populations. Syndemics are defined as synergistic epidemics: clusters of co-occurring conditions that interact biologically and socially, magnifying their cumulative health impacts in the context of structural inequity (4). Coined by Merrill Singer, a *syndemic* refers to the co-occurrence of two or more adverse health conditions that interact exponentially under conditions of social inequality and systemic neglect, producing excess burden beyond the sum of individual effects (5). The health impact of poverty-violence-health syndemic cannot be overstated. The bidirectional relationship between physical and mental health is well-established, particularly among individuals facing chronic adversity (6). For example, those with chronic medical conditions (e.g., cardiovascular disease, metabolic conditions, cancer) are at increased risk for depression or other psychological conditions, and vice versa, impacting illness severity and treatment outcomes (7, 8). Chronic physical and mental health conditions can significantly reduce quality of life and daily functioning. These challenges often reinforce the cycle of the syndemic through factors such as high treatment costs, disability, or limited ability to work (9). Further, healthcare and social services largely operate separately in silos, insufficiently equipped to address the broader social determinants that drive health disparities.

This Perspective argues that a syndemic-informed approach represents more than an integrative framework; it is a structural reorientation of healthcare delivery. Whereas existing models such as integrated care, trauma-informed practice, and SDOH screening often address co-occurring risks in parallel, a syndemic framework centers the *interacting* biological, psychological, and structural mechanisms that amplify disease burden. We propose that improving outcomes in marginalized populations requires shifting from additive models of care coordination toward system designs that intervene simultaneously on clustered conditions and their shared upstream drivers. We argue that health equity cannot be achieved through coordination alone; it requires system redesign that targets the interacting mechanisms producing disease clustering.

## The poverty-violence-health syndemic

When examined individually, poverty imposes chronic stress through food insecurity, housing instability, unemployment, and limited healthcare access, compromising both physiological resilience and well-being (5). In 2023, approximately 12% of the U.S. population lived in poverty, with rates exceeding 19% among Black Americans (1). In turn, violence, whether interpersonal or structural, including mass incarceration, racialized policing, and systemic disinvestment—further escalates mental health risks, including depression, posttraumatic stress disorder (PTSD), and substance use disorders (10). Black Americans are more than twice as likely to die by firearm homicide compared to White Americans (11), and Black women are significantly more likely to experience intimate partner violence (12). The combined effects of poverty and violence create barriers to health-supportive behaviors (e.g., avoidance), reduce engagement in preventive care, and thus exacerbate chronic illnesses. Importantly, these disparities are socially produced and institutionally sustained through the systemic earned mistrust of healthcare systems by marginalized groups, not individual failings. This mistrust is historically rooted in documented medical exploitation and neglect, including the Tuskegee Syphilis Study, the non-consensual use of Henrietta Lacks’ cells, forced sterilization of women of color, and persistent racial disparities in pain management and maternal mortality.

**Figure 1:**
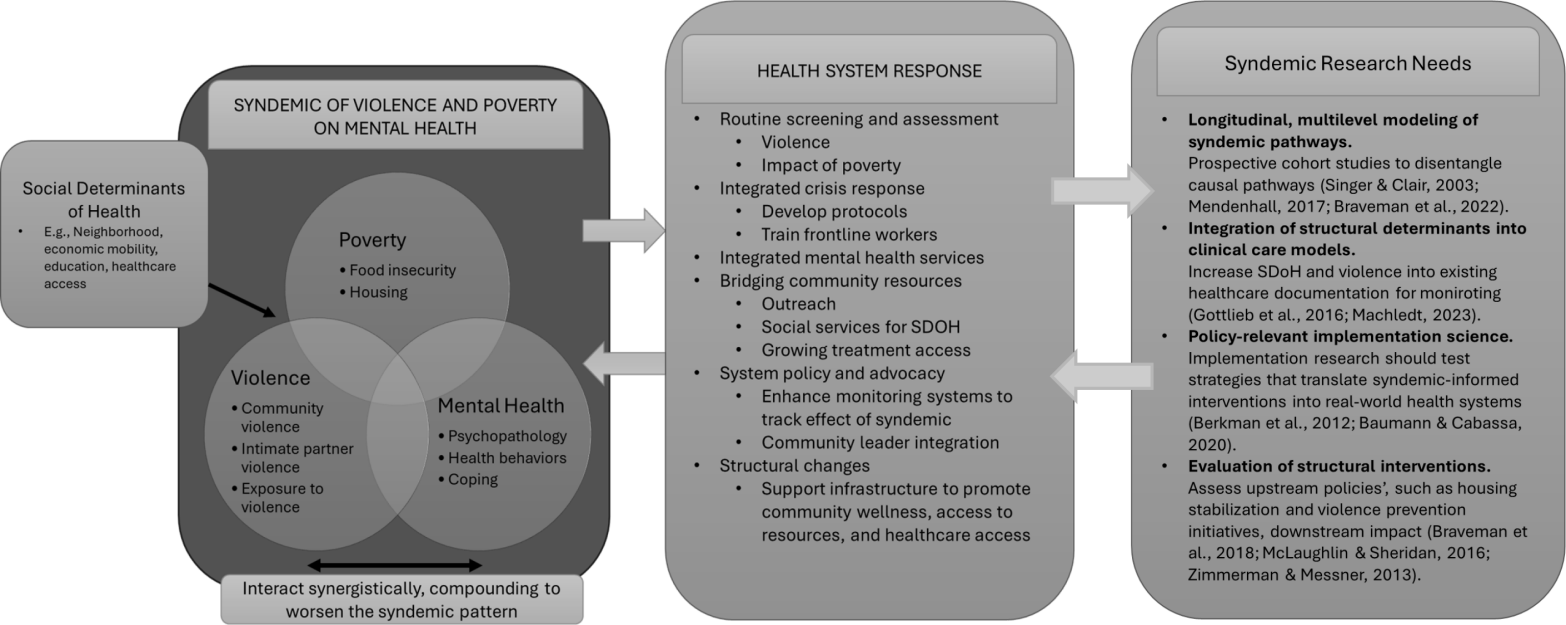
Conceptual model of syndemics of poverty, violence and mental health.

The syndemic framework offers a critical lens to examine the mutually reinforcing burdens of poverty, violence, and health disparities, particularly in structurally marginalized communities (**Figure 1**).

The socioecological model highlights how an individual’s experience is embedded within and shaped by their context^4^. In the United States, structural discrimination perpetuates disparities in exposure to poverty, violence, and adverse health outcomes. Black Americans are incarcerated at nearly five times the rate of White Americans (13), and incarceration itself is associated with elevated risk of chronic disease, psychiatric disorders, and premature mortality (14). However, it is structural disadvantage, rooted in historical disinvestment, that promotes this violence disparity for marginalized groups. Take, for instance, how people in poverty and marginalized groups are more likely to be incarcerated, which has a direct relationship to risk of violence^6^. Ultimately, it is vital to see that these disparities are not simply individual or behavioral or affect someone in isolation, but are driven by systemic disadvantage, including entrenched poverty and community disinvestment, all of which is important to explore to reach sustainable solutions.

This syndemic then cascades to individual health, with COVID-19 serving as a grim example of how disparities worsen within global crises, disproportionately impacting marginalized communities in mental health risk and access to care (15). Similarly, high rates of untreated mental illness—compounded by under-resourced public health infrastructure, and criminalization of psychiatric symptoms—create a landscape in which suffering is normalized and invisible. Nearly half of U.S. adults with a diagnosable mental health condition receive no treatment each year, with Black, Hispanic, and Asian adults significantly less likely to access outpatient mental health services compared to White adults. Using nationally representative U.S. survey data from the early COVID-19 pandemic, Williams and Vermund (16) found that 41–44% of Black and Hispanic adults reported depressive symptoms, compared with approximately 37% of White adults, based on self-reported mental health measures included in their syndemic analysis. (16). Further, treatment access disparities exist with Hispanic, Asian and Black female adults especially having greater difficulty finding suitable, affordable, and culturally responsive care in comparison to their White counterparts. This produces understandable individual and community-level mistrust in systems further reducing help-seeking behavior.

## Healthcare implications

A central limitation of current healthcare approaches is the growing mismatch between risk identification and response capacity. Health systems increasingly screen for social determinants, trauma exposure, or violence risk, yet often lack the institutional infrastructure, cross-sector partnerships, or reimbursement mechanisms to intervene on these findings. Screening without actionable pathways may inadvertently burden patients, heighten distress, and shift responsibility onto individuals for structural problems. Addressing syndemic conditions therefore requires not only detection of risk but system-level redesign that ensures identified needs can be met.

The burden of navigating fragmented services, waiting lists, and culturally incongruent care models further diminishes health agency. If patients can overcome these systemic barriers (e.g., insurance status, transportation, mistrust), patients experiencing syndemic-level stressors present in frontline healthcare settings, including emergency departments, primary care, and inpatient units, often with overlapping medical and mental health needs. Yet healthcare systems largely operate in silos, limiting their capacity to address the upstream social determinants that amplify disease burden (17, 18). A syndemic-informed approach recognizes that effective healthcare must integrate behavioral health, trauma-informed care, and structural competency to improve outcomes for marginalized populations.

## Benefits of a syndemic-informed approach

Existing integrated and trauma-informed care models have advanced patient-centered practice by improving coordination and clinical responsiveness. However, these approaches typically operate within existing institutional structures and focus on managing co-occurring conditions rather than disrupting the mechanisms that cause them to cluster. A syndemic-informed approach extends beyond coordination to explicitly address interaction effects between conditions (e.g., violence exposure intensifying chronic disease progression) and therefore necessitates structural interventions, cross-sector accountability, and prevention-oriented resource allocation (19). This distinction shifts healthcare from a reactive model of managing multimorbidity to a proactive model of reducing disease amplification.

Improving access to preventive medical care is equally critical within a syndemic-informed framework. Evidence suggests that Medicaid expansion, Federally Qualified Health Centers, and community-based primary care models increase preventive screening, chronic disease management, and behavioral health integration among low-income populations. Co-located services, mobile health clinics, and integrated behavioral health within primary care reduce logistical barriers while enhancing continuity of care. Expanding reimbursement for preventive services and embedding care navigators within healthcare teams can further reduce fragmentation and promote early intervention.

## Recommendations for healthcare practice, policy, and research

To operationalize a syndemic-informed healthcare system, three reforms should be prioritized. First, screening must be linked to response infrastructure, including embedded care navigators, community partnerships, and reimbursable referral pathways. Second, financing and data systems must support cross-sector coordination so that healthcare, housing, violence prevention, and social services can jointly address shared determinants of health. Third, workforce training should expand beyond cultural competence to include structural and syndemic competence, equipping providers to recognize interacting risks and intervene at multiple ecological levels. Prioritizing these reforms is essential because they address foundational system constraints that currently prevent implementation of equity-oriented care models.

### Practice

Healthcare systems should expand SDOH screening to include trauma, violence exposure, and mental health comorbidities, using trauma-informed, culturally responsive approaches. Co-locating behavioral health within primary and emergency care, alongside integrated case management and community referral pathways, ensures that care is responsive to syndemic-level stressors. Training providers in structural competency, trauma-informed care, and implicit bias supports holistic patient care, while adopting strengths-based approaches fosters engagement and resilience.

Strengths-based approaches may include incorporating peer navigators with lived experience, partnering with community health workers embedded in neighborhoods, partnering with faith-based organizations, utilizing narrative-based interventions that center patient resilience, and engaging families as collaborative partners in care planning.

### Policy and system-level strategies

Intersectoral partnerships between healthcare, social services, housing, and violence prevention organizations can reduce patient burden and improve care continuity. Data systems that track intersecting exposures to poverty, violence, and health outcomes can guide resource allocation, quality improvement, and risk stratification. Engaging individuals with lived experience in advisory roles ensures that care and policy decisions reflect community needs.

### Research

Incentivizing research in equity-oriented care through value-based funding mechanisms. Using a stepped approach—starting with qualitative methods to capture lived experience (e.g., exposure to violence, impact of poverty on healthcare access) and moving to implementation studies to evaluate integrated care models—ensures programs both reflect patient needs and are delivered effectively. Specifically, assessing the unique implementation needs and opportunities for distinct healthcare settings, such as hospitalization being a critical window for violence prevention (20). Further, including structural determinants, intersectionality, and the multilevel effects of syndemic exposures as covariates in health outcomes research, could elucidate how they influence treatments.

### Community and public health

Investment in grassroots wellness infrastructure and peer-led initiatives strengthens protective networks within marginalized communities. Cross-training community health workers in physical health, mental health first aid, trauma screening, and care navigation enhances their role as bridges between formal systems and community members. Upstream preventive interventions—targeting housing, employment, education, and neighborhood safety—should be framed as integral to holistic healthcare delivery under a syndemic-informed model.

## Conclusion

Addressing the syndemic of poverty, violence, and health requires a paradigm shift in healthcare—from treating isolated diseases to confronting the structural and social conditions that drive inequities. Integrating behavioral health, trauma-informed practices, and structural competency into healthcare settings, coupled with intersectoral partnerships, community engagement, and research that centers lived experience, can transform health systems to more effectively serve marginalized populations to mitigate the intertwined burdens of poverty, violence, and health disparities.

## Data Availability

The original contributions presented in the study are included in the article/supplementary material. Further inquiries can be directed to the corresponding author.
